# Analysis of heterogeneity in T_2_-weighted MR images can differentiate pseudoprogression from progression in glioblastoma

**DOI:** 10.1371/journal.pone.0176528

**Published:** 2017-05-17

**Authors:** Thomas C. Booth, Timothy J. Larkin, Yinyin Yuan, Mikko I. Kettunen, Sarah N. Dawson, Daniel Scoffings, Holly C. Canuto, Sarah L. Vowler, Heide Kirschenlohr, Michael P. Hobson, Florian Markowetz, Sarah Jefferies, Kevin M. Brindle

**Affiliations:** 1 Department of Biochemistry, University of Cambridge, Cambridge, United Kingdom; 2 Cancer Research UK Cambridge Institute, University of Cambridge, Li Ka Shing Centre, Cambridge, United Kingdom; 3 Cambridge Clinical Trials Unit, Cambridge University Hospitals NHS Foundation Trust, Cambridge, United Kingdom; 4 Department of Radiology, Addenbrooke’s Hospital, Cambridge, United Kingdom; 5 Battock Centre for Experimental Astrophysics, Cavendish Laboratory, University of Cambridge, Cambridge, United Kingdom; 6 Department of Oncology, Addenbrooke’s Hospital, Cambridge, United Kingdom; George Washington University, UNITED STATES

## Abstract

**Purpose:**

To develop an image analysis technique that distinguishes pseudoprogression from true progression by analyzing tumour heterogeneity in *T*_2_-weighted images using topological descriptors of image heterogeneity called Minkowski functionals (MFs).

**Methods:**

Using a retrospective patient cohort (*n* = 50), and blinded to treatment response outcome, unsupervised feature estimation was performed to investigate MFs for the presence of outliers, potential confounders, and sensitivity to treatment response. The progression and pseudoprogression groups were then unblinded and supervised feature selection was performed using MFs, size and signal intensity features. A support vector machine model was obtained and evaluated using a prospective test cohort.

**Results:**

The model gave a classification accuracy, using a combination of MFs and size features, of more than 85% in both retrospective and prospective datasets. A different feature selection method (Random Forest) and classifier (Lasso) gave the same results. Although not apparent to the reporting radiologist, the *T*_2_-weighted hyperintensity phenotype of those patients with progression was heterogeneous, large and frond-like when compared to those with pseudoprogression.

**Conclusion:**

Analysis of heterogeneity, in *T*_2_-weighted MR images, which are acquired routinely in the clinic, has the potential to detect an earlier treatment response allowing an early change in treatment strategy. Prospective validation of this technique in larger datasets is required.

## Introduction

The commonest primary malignant brain tumour, glioblastoma, is a devastating disease with a progression free-survival of 15% at 1 year.[1] Maximal debulking surgery and radiotherapy, with concomitant and adjuvant temozolomide, is the standard of care[2] but is associated with pseudoprogression. This describes false-positive progressive disease within 6 months of chemoradiotherapy, typically determined by changes in contrast enhancement on *T*_1_-weighted MR images, representing non-specific blood-brain barrier disruption.[3] Pseudoprogression confounds response assessment and may affect clinical management. An imaging technique that reliably differentiates responders from non-responders would allow an early change in treatment strategy with prompt termination of ineffective treatment and the option of implementing novel therapies.[4] To achieve this, we describe a method that is simple to implement, requires little computational effort, is intuitive to interpret[5] and only requires *T*_2_-weighted images that are acquired routinely during patient follow-up and which more accurately detect glioblastoma infiltration than contrast-enhanced *T*_1_-weighted images.[6;7] This is because glioblastoma cell infiltration, which can cause hyperintensity in *T*_2_-weighted images, does not necessarily result in the blood-brain barrier disruption required for detection in contrast-enhanced *T*_1_-weighted images. Analysis of *T*_2_-weighted images to determine treatment response is therefore now routine in the clinic, although the detail of how to determine response from these images is a topic of ongoing research.[2;8–11] Our approach exploits the fact that tissue morphology can be a sensitive marker of underlying tissue biology[12] and that morphological information can be extracted from an MR image[13] using image descriptors called Minkowski functionals (MFs). MFs can be used to parameterize the heterogeneous distribution of hyper- and hypointense foci in *T*_2_-weighted tumour images and have been shown recently to be capable of detecting treatment response through changes in the size and distribution of these foci in pre-clinical MR images, including *T*_2_-weighted images.[13;14] Previously applied to describe the complex morphology of galaxies,[15] MFs can capture underlying tumour heterogeneity not apparent to the reporting radiologist. The purpose of this study was to extend the use of MFs to clinical MRI data and develop an imaging technique that can distinguish pseudoprogression from true progression by analysing heterogeneity in *T*_2_-weighted MR images.

## Methods

### Participants

The UK National Research Ethics Service approved the retrospective and prospective arms of the study (written or verbal informed consent to participate in this study, which used de-identified data, was not a requirement by the UK National Research Ethics Service). Eligible patients (S1 Table) were those with recently diagnosed glioblastoma who underwent chemoradiotherapy according to the Stupp regimen on an intention-to-treat basis.[2] All patients completed a 6 week radiotherapy course of 60 Gy in 30 fractions, following institutional protocol,[16] except for one patient who received 60 Gy to the planning target volume and 80 Gy to the gross tumour volume. Patients entered into drug trials were excluded and bevacuzimab was not prescribed in keeping with European recommendations.[17] *T*_2_-weighted images were obtained at baseline (post-surgical in the week preceding chemoradiotherapy) as well as at 4 weeks, 4 months and 7 months following completion of chemoradiotherapy.

### MR acquisition and image processing

Axial *T*_2_-weighted images were acquired on General Electric scanners (two 1.5 T and a 3 T Signa EXCITE; a 1.5 T and a 3 T HDx). On the 1.5 T scanners spin echo pulse sequence parameters were: repetition time (TR) 5.000–6.260 s, echo time (TE) 0.096–0.108 s, with a single excitation and echo train length of 24. Spin echo parallel imaging (ASSET) was performed on the 3 T scanners with TR 4.000–6.080 s, TE 0.099–0.101 s, using a single excitation with an echo train length of 32. The centre of k-space was acquired in the middle of the echo train length. The receiver bandwidth was 81–89 kHz and 122 kHz for the 1.5 T and 3 T scanners respectively. The field of view was either 220 x 220 mm or 240 x 240 mm and the slice thickness was 6 mm, interspaced with a 1 mm gap. On the 1.5 T scanners the matrix was 320 x 256 or 384 x 384 or 384 x 256 and zero-filled to 512 x 512. On the 3 T scanner the matrix size was 512 x 384 and was zero-filled to 1024 x 1024. In the prospective cohort some scans were also performed on a 1.5 T General Electric Discovery MR450 and in a single case a TR of 3.563 s was used.

**Fig 1:**
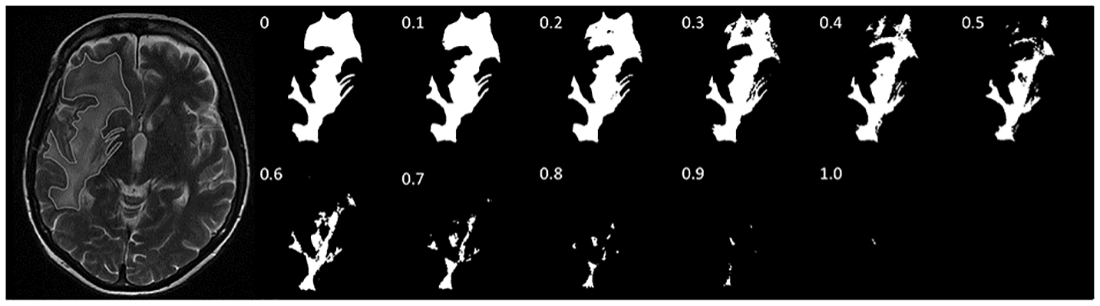
Grey-scale thresholding of a region of interest. A segmented region of interest (ROI) displayed as a binary combination of black and white pixels at 11 different grey-scale thresholds. Each of these black and white images can be characterized by the three 2D MFs; area, perimeter and genus.

Pathological *T*_2_-weighted hyperintense regions of interest from each slice containing tumour were segmented manually by a neuroradiologist (T.C.B.) and normalized for signal intensity using in-house MATLAB (Mathworks, Natick, US) code (available below).[14] Cyst-like structures larger than 1 cm in diameter were excluded to prevent analysis of surgical resection cavities or large necrotic cavities rather than tissue. Tumour images were converted into binary datasets by thresholding, where ten threshold steps were chosen to sample the grey scale, giving 11 thresholded images per slice (pre-clinical studies [13;14] have shown that there is no benefit in using more than 11 thresholds). Pixels were assigned as either black (below threshold) or white (above threshold) (Fig 1). The faces, edges and vertices of the designated (white) pixels were used to calculate the three 2D MFs (area, perimeter and genus),[5] which were normalized to the total number of pixels in the segmented image.[14] The summed pixel faces give the area, the summed pixel edges at the boundary between black and white pixels give the perimeter, and the number of regions of connected white pixels minus the number of completely enclosed regions of black pixels gives the genus. Size features (total area and perimeter) and signal intensity features (mean, minimum, maximum and standard deviation; all normalized to contralateral white matter) were also obtained since these are associated with treatment response in glioblastoma,[18;19] and were acquired with minimal additional computational effort. Thirty-two MF, two size and four signal intensity features were obtained for each multi-slice image (detailed in Table 1).

**Table 1:** Definitions of features.

Variable	Definition	Mathematical Formulae^a^
**Minkowski functionals**		
** Area**	Summed designated (white) pixels.	*A*_*i*_ = *p*_*i*_
** Perimeter**	Summed pixel edges at the boundary between black and white pixels.	*U*_*i*_ = -4*p*_*i*_ + 2*e*_*i*_
** Perimeter 1**^b^	Perimeter at grey-scale threshold 1 (i.e. all pixels are white). *The edge length (or contour) of the segmented region of interest*.	*U*_0_ = -4*p*_0_ + 2*e*_0_
** Genus**	Number of regions of connected^c^ white pixels minus the number of completely enclosed regions of black pixels.	*χ*_*i*_ = *p*_*i*_* − e*_*i*_ + *v*_*i*_
**Size**		
** Total Area**	Summed area at grey-scale threshold 1 (i.e. all pixels are white) for all slices of the tumour.	$TA_{k}=\sum_{j=1}^{J}{\left(A_{0}\right)}_{j}$
** Total Perimeter**	Summed perimeter at grey-scale threshold 1 for all slices of the tumour. *The summed edge length (or contour) of the segmented regions of interest from all slices of the tumour*.	$TP_{k}=\sum_{j=1}^{J}{\left(U_{0}\right)}_{j}$
**Signal intensity**		
** Mean**	The mean raw signal intensity of all pixels from the segmented regions of interest from all slices of the tumour.	$\mu_{k}=\frac{1}{TA_{k}}\sum_{p=1}^{TA_{k}}SI_{P}$
** Standard Deviation**	The standard deviation of the raw signal intensity of all pixels from the segmented regions of interest from all slices of the tumour.	$\sigma_{k}=\sqrt{\frac{1}{TA_{k}}\sum_{p=1}^{TA_{k}}\left(SI_{P}-\;\mu_{k}\right)}$
** Maximum**	The maximum raw signal intensity of all pixels from the segmented regions of interest from all slices of the tumour.	$\;\;\;\wedge_{k}=\;SI_{\left(TA_{k}\right)}$
** Minimum**	The minimum raw signal intensity of all pixels from the segmented regions of interest from all slices of the tumour.	$\vee_{k}=\;SI_{\left(1\right)}$

^a^ Where *p*_*i*_, *e*_*i*_, and *v*_*i*_ are the numbers of pixels, edges, and vertices at grey-scale threshold *i*, respectively; *j* is the number of image slices through the tumour; *k* is the individual tumour; *SI*_*p*_ is the raw signal intensity for pixel *p*.

^**b**^ A segmented region of interest was displayed as a binary combination of black and white pixels at 11 different grey-scale thresholds (Fig 1). Perimeter 1 in Table 1 is the perimeter at grey-scale threshold 1, from a total of 11. Eleven different grey-scale thresholds were also used for genus. Ten different grey-scale thresholds were used for area because the 1^st^ value ≡ 1 when normalized and so was excluded.

^**c**^ Two pixels are connected if they are nearest neighbors or next-nearest neighbors to each other.

### Feature estimation

**Fig 2:**
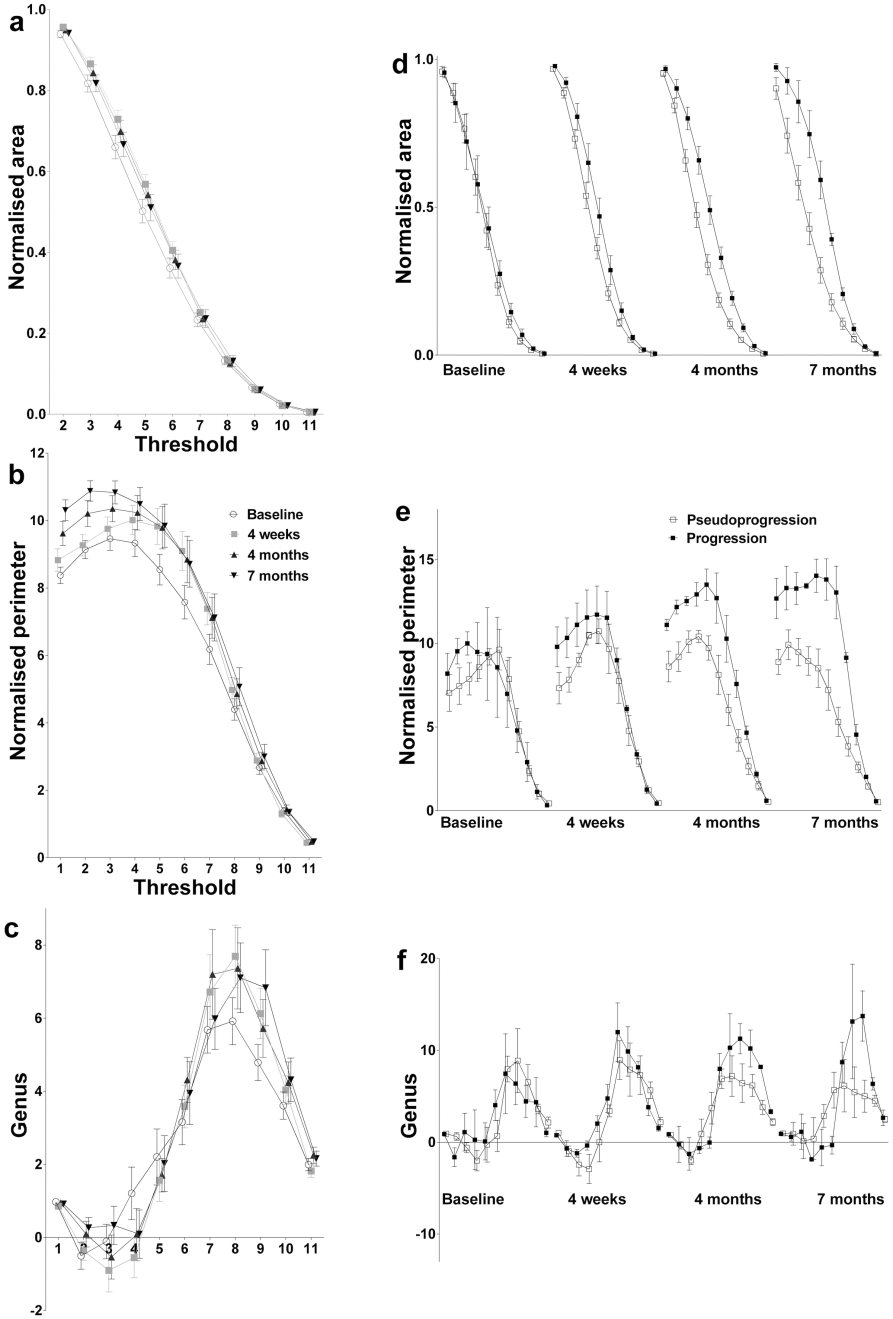
MFs of the retrospective patient cohort. Spectral representations of MFs (mean ± standard error) plotted as a function of grey-scale threshold using blinded (a-c) and unblinded (d-f) data from the retrospective patient cohort. The more heterogeneous the regions of interest, the higher the normalized perimeter value; and the further the genus value is from unity.

This involves exploration of image analysis features to determine whether they are suitable biomarkers to test a clinical hypothesis.[20] Blinded to patient outcome and treatment response status, MFs from the retrospective cohort were investigated for the presence of outliers and potential confounders by principal component analysis using SIMCA-P+ v12 (Umetrics, Kinelon, US). Hotelling T2 (a multivariate generalisation of Student's *t*-test) was set at the 0.05 significance threshold. A linear mixed model in R (version 2.11.1, http://www.r-project.org) was used to examine whether MFs detected an early increase in heterogeneity after chemoradiotherapy, regardless of treatment response status. Here, the MF values describing the heterogeneous distribution of hyper- and hypointense foci in T_2_-weighted images before chemoradiotherapy were compared to the MF values derived from *T*_2_-weighted images after chemoradiotherapy (4 weeks, 4 months and 7 months later; all time points were used for the analysis). The nlme package was used, which allows for nested random effects.[21] A hierarchical model of time, nested within threshold, nested within patient, was used to model the covariance structure of the data. Time and threshold were treated as categorical factors and fixed effects. An interaction term between time and threshold was also considered as a fixed effect to investigate if the effect of time was different at each threshold, as this possibility was suggested by plots of the data (Fig 2a–2c). Random effects were also considered for time and threshold. Patient effects were designated as random effects, as the effects of individual patients were not of interest (the patients were considered as a random sample of the overall population of patients), along with threshold nested within patient. Modeling incorporated potential confounders as covariates, which included age, surgical status (resection (i.e. debulking) or biopsy), Karnofsky performance status, location, treatment compliance and pre-operative tumour size. These parameters are known to be the most significant survival predictors.[1;22–24] A neuro-oncologist (S.J.) assigned all clinical categories and assessed steroid use and a neuroradiologist (D.S.) calculated the pre-operative tumour size as the product of the two largest orthogonal dimensions on *T*_1_-weighted post-contrast images. If all 4 time-points were not available, the available time points were used rather than exclude the patient. Goodness-of-fit was assessed using a Q-Q plot and a histogram of the residuals and by using a scatterplot of the residuals plotted against the fitted values.

### Response outcome designation

After unblinding, patients were designated response outcomes (S1a Fig) using the Response Assessment in Neuro-Oncology (RANO) criteria (including steroid use criteria)[8] as the basis for the reference standard. However, incorporating more recent evidence, we also included in the pseudoprogression group patients who had an increasing enhancing lesion at 4 weeks following chemoradiotherapy, which continued to increase at 4 months but improved or stabilized at 7 months,[25] and patients who had an increasing enhancing lesion at 4 months following chemoradiotherapy, which stabilized or improved at 7 months.[26] This modified approach incorporates pseudoprogression that occurs or extends beyond 3 months,[27–30] when a change of treatment is often considered.[31] In light of the importance of clinical features in the MacDonald[3] and RANO criteria, patients with stable imaging but worsening neurological features that improved subsequently without an increase in steroid use were also assigned as pseudoprogressors. The final outcome assessments were validated using a univariate log-rank test (Mantel-Cox) and plotted using the Kaplan-Meier method (S1c Fig).

### Feature selection, classification and evaluation

The aim of this study was to differentiate progression and pseudoprogression as early as possible i.e. at the earliest time point when an enlarging MRI-enhancing lesion was seen, which was either at 4 weeks in some cases or at 4 months in others. This is the critical period when a clinician must decide whether to continue with the current patient management plan or to change it. The features derived from *T*_2_-weighted images from these two earliest time points alone were used in susbsequent analyses.

*Feature selection* selects the most discriminant features and reduces the number of variables to optimise classification. The MF, size and signal intensity features selected by *t*-tests underwent support vector machine (SVM) supervised analysis using MATLAB software with LIBSVM code.[32] Parameters were optimized using leave-one-out cross-validation. The SVM model was then built using these optimized parameters and the training set tested using leave-one-out cross-validation. The accuracy, sensitivity and specificity of the model were calculated using the Wilson score method.[33] Classification performance was evaluated further by a receiver operator characteristic analysis.[34] SVM decision values were tested as predictors of survival using a multivariate Cox-proportional hazards regression model using XLSTAT 2012 (Addinsoft SARL, Paris, France). A prospective dataset (S1b Fig) was then evaluated using the classifer constructed from the training dataset. To further validate that there might be an underlying *T*_2_-weighted hyperintensity phenotype that can distinguish progression and pseudoprogression, all the original variables from the training and test datasets were analysed using a different mathematical strategy. The training dataset underwent feature selection using Random Forest[35] and machine learning classification using Lasso (gradient ascent optimization and the Newton-Raphson algorithm[36]) and subsequently the classifier was applied to the test dataset (all analyses were performed in R).

### Comparison with vasogenic oedema and radiation necrosis

To understand better what processes the selected features were reporting in those patients with progression or pseudoprogression, comparison was made with *T*_2_-weighted images of vasogenic oedema and biopsy-proven radiation necrosis. The *T*_2_-weighted images were segmented, MFs calculated and analysed using principal component analysis and then classified using the classifier constructed from the training dataset.

### Statistical overview

Unless stated otherwise, statistics were performed using Graphpad Prism 6 (La Jolla, US). Patient characteristics were compared using unpaired 2-tailed Student’s *t*-tests, 1-way ANOVA (Šídák’s multiple comparisons and Brown-Forsythe standard deviation tests), 2-tailed Fisher-Freeman-Halton (contingency tables larger than 2 x 2, and small expected frequencies within cells; Stata 11, College Station, US) or Fisher’s exact tests. Significance was set at *P* < 0.05. For parametric methods, normality was assessed using the D’Agostino-Pearson omnibus test.

### Freeware

MATLAB code to generate the features is freely available at http://mathworks.com/matlabcentral/fileexchange/62674-mrmf. R or MATLAB code to generate an SVM classifier is already freely available[32].

## Results

### Feature estimation

Fifty eligible patients were used for a retrospective training cohort (S1 Table). Tumour images were converted into binary datasets by thresholding (Fig 1) and the MF parameters were calculated as described previously[13] and in the methods section. The population of MFs acquired at 3 T differed to those obtained at 1.5 T, and contained outliers with high MF values (S2 Fig). Downsampling the larger data matrix obtained at 3T to the size of the 1.5T data matrix did not resolve the difference in MF parameters. Therefore, 3T data were excluded from all subsequent analyses (from 179 scans, 38 were excluded in the linear mixed model). The MF parameters from the blinded data showed that there was an increase in tumour heterogeneity in the *T*_2_-weighted images following chemoradiotherapy, as evidenced by an increase in the genus and perimeter values at some thresholds (Fig 2a–2c). This was confirmed using a linear mixed model, which provided a robust means to incorporate the intrinsic relatedness of each patient’s entire dataset and to allow for missing data (S2 Table). We also examined potential confounders which showed that few other covariates contributed to the final models and these were much less statistically significant than MFs (S2 Table). An increase in heterogeneity was anticipated since in irradiated tissue there is MRI-detectable microscopic tissue damage,[37] peritumoural tissues show increased diffusivity in MR images as early as three weeks from treatment initiation,[38] and there is increased vascular heterogeneity (measured as changes in vessel permeability) within two months of treatment completion.[39] Also studies in a rodent tumour model showed an increase in heterogeneity, assessed using MFs, in *T*_2_-weighted images following treatment.[14] Neuroradiology review found that there was no apparent increase in heterogeneity that was visible by eye, consistent with the limited human appreciation of complexity in an image.[40]

The retrospective training dataset was unblinded, treatment response assigned (24% pseudoprogression, 32% progression), and the earliest time point when an enlarging MRI-enhancing lesion occurred was recorded (S3 Table). To demonstrate that the modified RANO[8] criteria used here gave appropriate categorization of progression or pseudoprogression, a survival analysis was performed (S1c Fig), which supported the assigned treatment response status (*P* = 0.0006, χ^2^ = 12, 1 df; univariate log-rank test).

MFs derived from progressors diverged from pseudoprogressors consistent with an increase in image heterogeneity during progression (all 4 time points shown in Fig 2d–2f). This divergence was continuous with maximum divergence at 7 months. An increase in image heterogeneity is expected in glioblastomas undergoing progression following chemoradiotherapy because the tissue contains pseudopallisading necrosis and regions of microvascular proliferation with associated intratumoural microhaemorrhage, which are detectable by MRI.[20;41] In summary, feature estimation showed that MFs are suitable biomarkers to be used to test the hypothesis that progression and pseudoprogression can be detected at the earliest time point when an enlarging MRI-enhancing lesion was seen.

### Feature selection

**Fig 3:**
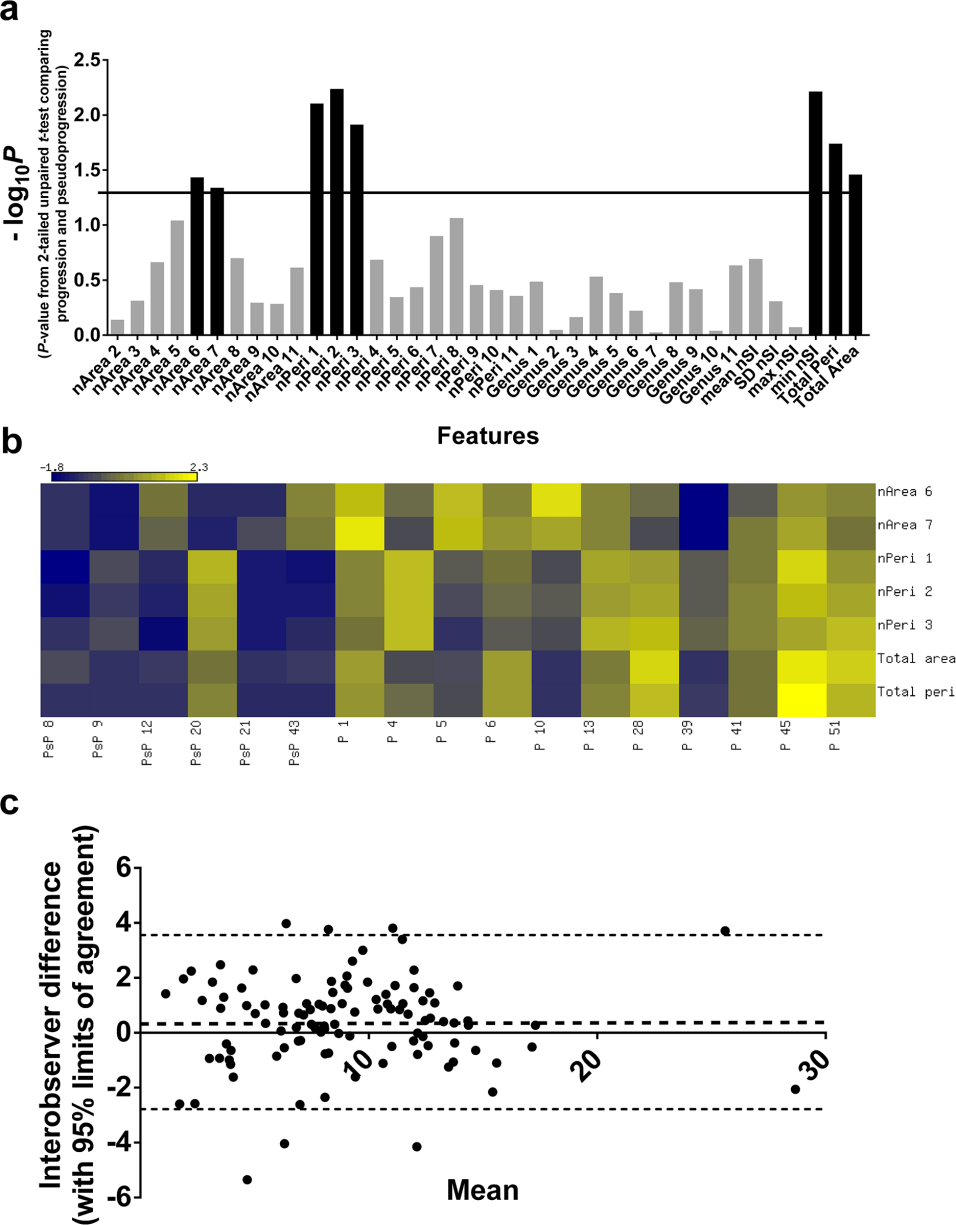
Training dataset feature selection. (**a**) Graph of -log_10_*P* values for all features derived by comparing progression and pseudoprogression datasets (2-tailed unpaired *t*-test). The significance threshold was set arbitrarily at *P* < 0.05 (horizontal line) and the selected features are shown as solid black bars. (Abbreviations: nArea, normalized area; nPeri, normalized perimeter; nSI, normalized signal intensity). (**b**) Heat map showing the selected features from the univariate scaled values of the MFs and size metrics used in the optimal SVM model for the training dataset. Dark blue represents the lowest values and yellow the highest values. Note that patient 20 was the only case of pseudoprogression assigned on clinical grounds in the study and was a false positive. (Abbreviations: PsP, pseudoprogression; P, progression). **(c)** Bland-Altman plot comparing the difference between two observer measurements, and the mean of the measurements. The bias was 0.4±1.6. The mean bias and the 95% limits of agreement of the interobserver difference are shown as dotted lines.

**Fig 4:**
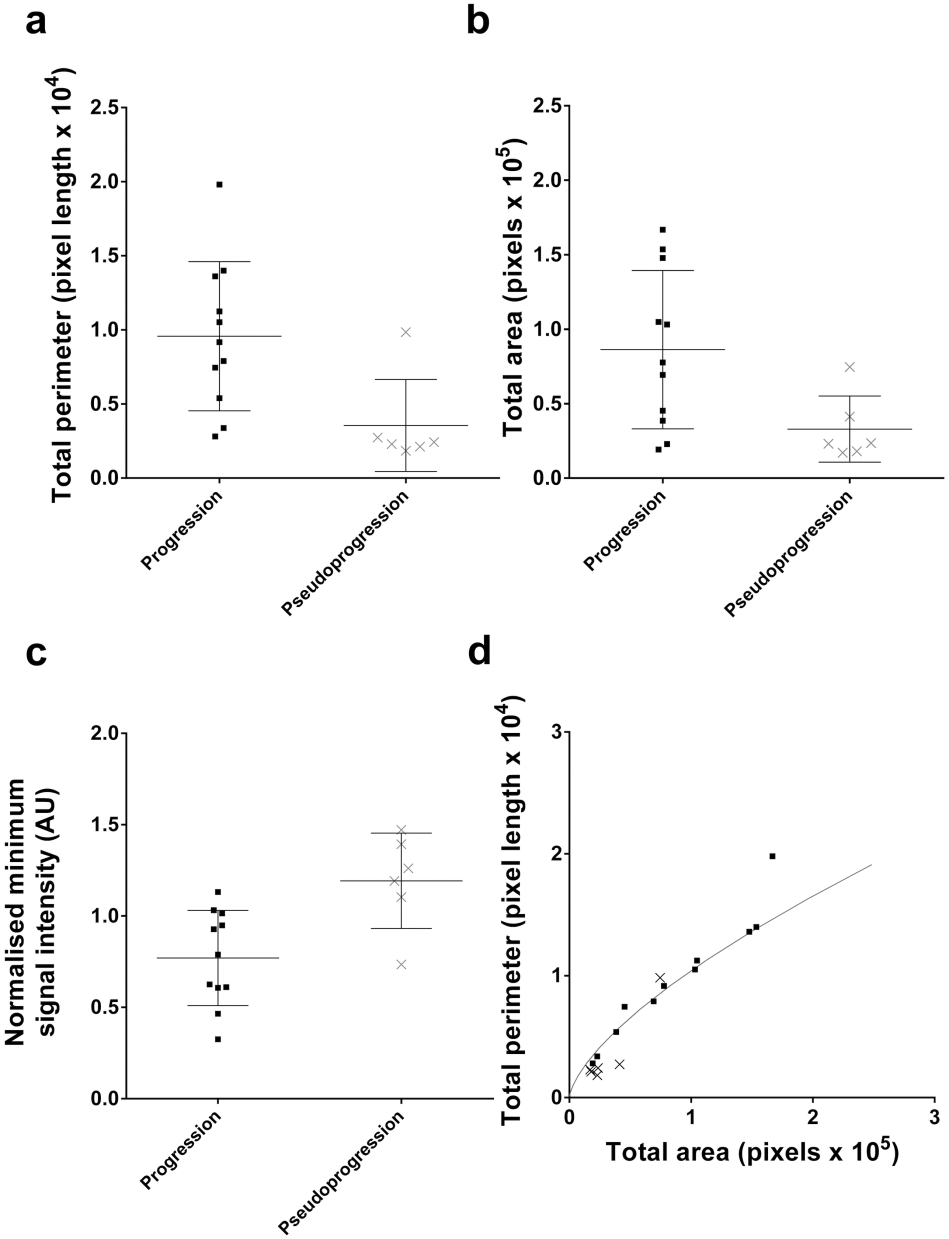
Training dataset size and signal intensity selected features. Plot of (**a**) total perimeter (*P* = 0.03, *t* = 2.3, 15 df) and (**b**) total area (*P* = 0.02, *t* = 2.6, 15 df) for patients with progression and pseudoprogression in the training data set. (**c**) Plot of the normalized minimum signal intensity (*P* = 0.006, *t* = 3.2, 15 df) for patients with progression and pseudoprogression in the training data set. (**d**) Relationship between total area and total perimeter for patients with progression (squares) and pseudoprogression (crosses) in the training data set. Total area is a surrogate metric of volume (a stack of slices summed together) and total perimeter is a surrogate metric of surface area (a stack of slice perimeters summed together). The solid line gives the relationship between volume and surface area for a sphere.

**Fig 5:**
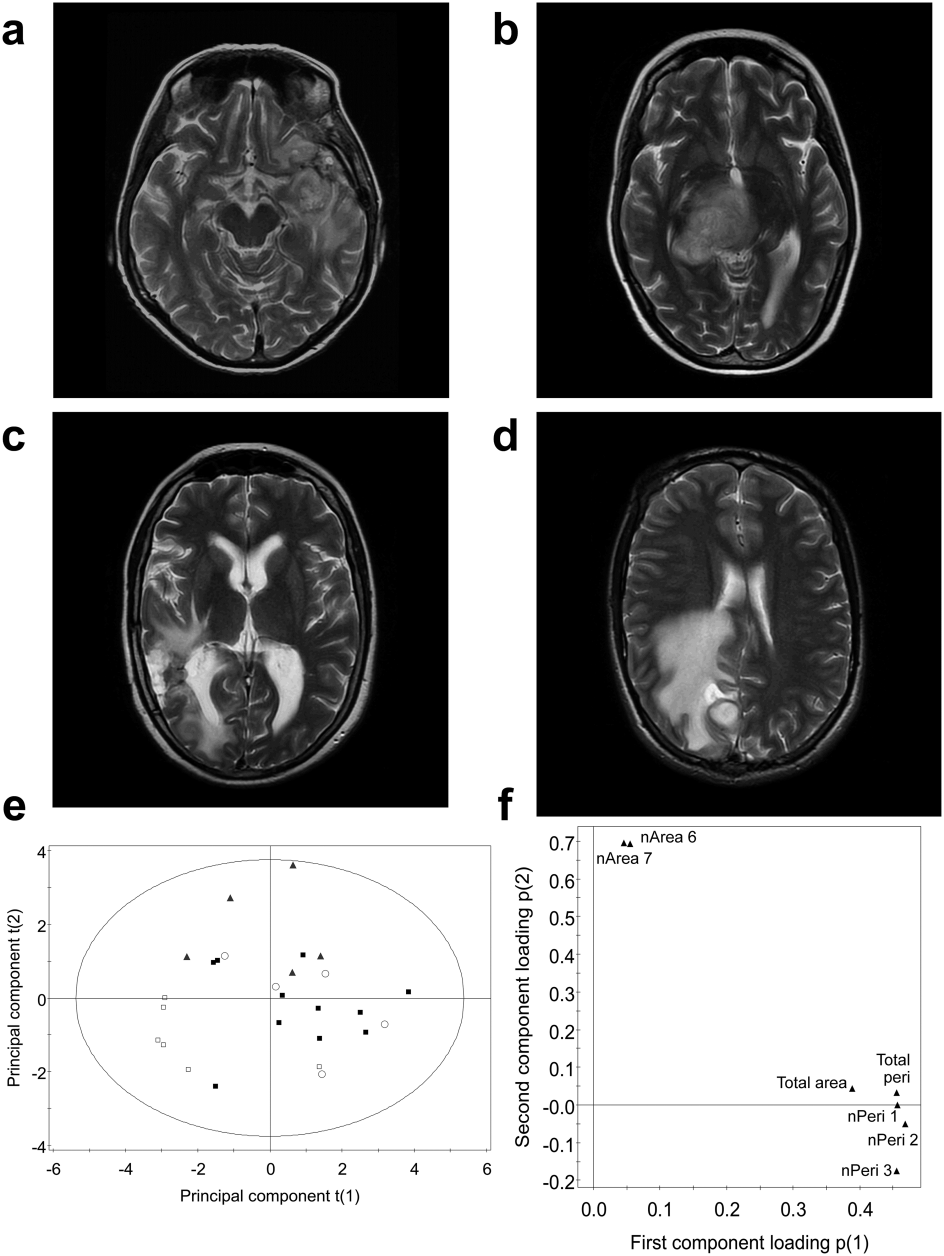
Model comparison with radiation necrosis and vasogenic oedema. *T*_2_–weighted axial images showing a patient with (**a**) progression, (**b**) pseudoprogression, (**c**) radiation necrosis, and (**d**) vasogenic oedema. Principal component score plot (**e**) for those patients with progression (filled black squares) and pseudoprogression (empty squares) from the training dataset, using the features selected for the original SVM model. Vasogenic oedema (triangle) and radiation necrosis (empty circle) were also plotted using the same set of selected features. All features were univariate-scaled. The displayed T2 Hotelling’s tolerance ellipse is set at the 0.05 significance level. The loading plot (**f**) corresponding to the principal component score plot, showed that the normalized perimeter and size features were positively correlated and separated pseudoprogression from progression and radiation necrosis.

To optimize classification, the number of features (MFs, size and descriptive statistics for signal intensity) were reduced by selecting, using unpaired *t*-tests, those individual features where there was a significant difference between progressors and pseudoprogressors at the earliest time point when an enlarging MRI-enhancing lesion was seen (Fig 3a). Tumours showing progression were larger than those showing pseudoprogression; the mean values of total area and perimeter were greater in progressors than pseudoprogressors (*P* = 0.03, *t* = 2.3, 15 df; *P* = 0.02, *t* = 2.6, 15 df respectively) (Fig 4a and 4b). The first threshold value of the normalized MF perimeter value is a measure of edge length, with progressors having a longer contour length-per-unit-area than pseudoprogressors. The relationship between total lesion area and perimeter is shown in (Fig 4d), where data points above the curve represent tumours with more surface area than would be expected for a sphere. Tumours showing progression were generally above or on the curve, consistent with a longer contour length-per-unit-area of *T*_2_-hyperintensity compared to areas of pseudoprogression (second-order polynomial curves for progression and pseudoprogression both gave R^2^ > 0.9; these two curves and the curve of a sphere were different from one another: *P* = 0.03, F = 3.1, 6 dfn, 21 dfd; extra sum-of-squares F test), and is compatible with progressors having a more irregular or frond-like shape than a spherical shape,[42–44] although this difference was rarely visible in the image (Fig 5a and 5b).

The only signal intensity metric that differed between progressors and pseudoprogressors was minimum signal intensity, which was higher in pseudoprogressors (*P* = 0.006, *t* = 3.2, 15 df) (Fig 4c).

### Classification

For the training dataset, heterogeneity (MF) alone (as well as heterogeneity and size features combined) allowed progressors and pseudoprogressors to be SVM-classified with slightly more accuracy than size alone [88% vs. 82%; accuracy is defined as (true positive + true negative)/(true positive + false positive +true negative + false negative); S4 Table], but the combination of heterogeneity and size also produced a slightly more clinically meaningful model than heterogeneity alone (S5 Table). The combined MF and size SVM model was therefore chosen for subsequent analysis (accuracy of 88%; receiver operator characteristic area under the curve of 0.9 after leave-one-out cross-validation (LOOCV); S4 Table). The raw values of the selected features used in this final SVM model are shown in Fig 3b.

### Reliability assessment

We examined the reliability of our processing pipeline. Manual segmentation was not a source of inter-user variability. The MF dataset produced with our feature extraction freeware after neuroradiologist segmentation was compared to a dataset produced after novice segmentation (T.J.L.; 30 minutes of training) and demonstrated 100% (12/12) classification concordance when tested using the original trained SVM model. There was also good interobserver agreement, demonstrated using the Bland-Altman limits of agreement method (Fig 3c).

There was no computational variability in selection and classification. Using the entire training and test MF datasets, the *t*-test selection and SVM (again using a radial basis function) classifier operation was independently repeated by a second operator (Y.Y.) using different freeware (R) and identical results were obtained as expected.

### Comparison with vasogenic oedema and radiation necrosis

The selected features that discriminated progression and pseudoprogression were extracted from the *T*_2_-weighted hyperintensity of 5 cases of biopsy-proven radiation necrosis (Fig 5c) obtained more than 1 year after radiotherapy, and surrounding 5 pre-treated brain abscesses (florid vasogenic oedema) (Fig 5d). Classification using the original trained SVM models labeled all cases as progressors (100%, 10/10). Principal component analysis showed that the hyperintensities in *T*_2_-weighted images of patients with radiation necrosis and progression were similar (Fig 5e), sharing a heterogeneous, large, and long contour length-per-unit-area phenotype (Fig 5f), whereas pseudoprogression had a distinct phenotype. The hyperintensity in *T*_2_-weighted images of patients with vasogenic oedema had a phenotype that was distinct from that seen in both progression and pseudoprogression. Therefore, the imaging phenotype of pseudoprogression was similar to neither vasogenic oedema nor radiation necrosis.

### Evaluation with a prospective dataset

Fifty-seven eligible patients were identified for the prospective cohort (data acquired between 2009 and 2012). After introduction of the 2010 RANO guidelines[8] many patients no longer underwent a 4-week post chemoradiotherapy MRI at our institution. Consequently, many patients were excluded when the information required for this study, including pseudoprogression status, could not be determined. During this period some patients were enrolled in a trial where carmustine wafers were introduced into the resection cavity during surgery. Since these wafers change perilesional signal intensity,[45] these patients were also excluded. Seven patients were analyzed subsequently in the test dataset (S1b Fig), which was classified (using the classifier constructed from the training dataset) with an accuracy of 86% (accuracy is defined as (true positive + true negative)/(true positive + false positive +true negative + false negative; S6 Table). The retrospective and prospective datasets were re-analyzed using a different mathematical strategy of Random Forest for feature selection followed by Lasso classification. The features selected from the training dataset were again a combination of MF and size features (normalized perimeter thresholds 1–3, 7 and 8 as well as total perimeter) and the same results were obtained (86% test dataset classification accuracy; accuracy is defined as (true positive + true negative)/(true positive + false positive +true negative + false negative; S7 Table), demonstrating that accuracy was not dependent on the selection and classification methods used.

## Discussion

MFs have been shown here to be sensitive feature descriptors that can be used to detect an early increase in heterogeneity in *T*_2_-weighted images of glioblastomas after chemoradiotherapy, regardless of treatment response status. When a retrospective training dataset was unblinded to treatment response, MFs from progressors showed that the images became increasingly heterogeneous over time when compared to pseudoprogressors. A clinically meaningful training dataset was developed using modified RANO criteria and supported by a survival analysis (S1c Fig).

Differences in MFs were demonstrated between progressors and pseudoprogressors at the earliest time point when an enlarging MRI-enhancing lesion is seen. Using *t*-test feature selection the most discriminant of these MFs were chosen for classification. Size and signal intensity descriptors were obtained from the *T*_2_-weighted images with minimal additional computational effort and also underwent *t*-test feature selection. Total area and total perimeter were selected features as tumours with larger hyperintense lesions in *T*_2_-weighted images were more likely to progress. Of the signal intensity descriptors, minimum signal intensity was selected as it was higher in pseudoprogressors than progressors. This may have been due to hemosiderin present in intratumoural microhaemorrhage associated with microvascular proliferation[20] or to increased cellularity in patients with progression.

A combination of heterogeneity (MFs) and size features produced the optimal SVM model, which classified progressors and pseudoprogressors with an accuracy of 88%, with a receiver operator characteristic area under the curve of 0.9 after leave-one-out cross-validation. The segmentation method was reliable as demonstrated by 100% interobserver classification concordance and it took a novice less than 15 minutes to segment any tumour.

A prospective test dataset was classified with an accuracy of 86%. A different strategy using Random Forest feature selection and Lasso classification gave the same result. The *T*_2_-weighted hyperintensity phenotype of progression remained, which was heterogeneous, large, with a long contour length-per-unit-area (compatible with being more frond-like) when compared to pseudoprogression.

Although this study used images obtained at 1.5 T, the commonest clinical magnetic field strength, imaging at 3 T is becoming increasingly common. The increase in *R*_2_* (effective transverse relaxation rate) at 3 T leads to a greater range of tissue relaxation times[46] and may have led to an increase in signal heterogeneity and the observed increase in MF values. An implication is that, as with other quantitative imaging techniques such as those applied to dynamic susceptibility contrast-enhanced (DSC) or arterial spin labelling perfusion imaging, volumetric imaging and 1H-magnetic resonance spectroscopy,[47–54] longitudinal follow-up should be performed at the same field strength. Another implication is that because MF values were higher at 3 T it is possible that heterogeneity feature descriptors would be more discriminant than at 1.5 T, however there was insufficient data to examine this hypothesis.

The pathology of pseudoprogression is poorly understood although it is thought to involve changes to the vascular endothelium and the blood-brain barrier that are associated with vasogenic oedema.[26,55] Pseudoprogression and radiation necrosis have also been postulated to represent a continuum of post treatment changes.[55] We have shown that the imaging phenotype, and therefore the underlying tissue biology[3,4] of pseudoprogression was similar to neither vasogenic oedema nor radiation necrosis although this needs to be interpreted with caution as the number of samples was small.

### Comparison to other studies

Other imaging approaches for distinguishing progression from pseudoprogression are being investigated. DSC perfusion imaging has shown considerable promise (ROC AUC 0.9, sensitivity 77–100%, specificity 75–86%; training datasets alone used) although the studies were also relatively small (14–25 patients for the training datasets).[25;27;31;56;57] Relative cerebral blood volume (rCBV) summary statistics have produced discrepant results,[25;27;57;58] which has motivated interval regional relative cerebral blood volume analyses.[27;56] Dynamic contrast-enhanced imaging has also been used (sensitivity 90%, specificity 83%; for the training dataset), however model-free parameters such as the initial area under the curve are semi-quantitative and time-consuming to determine and there are segmentation and co-registration limitations.[59] Diffusion imaging has also shown promise in distinguishing progression from pseudoprogression (ROC AUC 0.7–1.0, sensitivity 69–93%, specificity 69–100%, for the training datasets; sensitivity 75%, specificity 100% in a test dataset of 9 patients).[60–63] However, as with dynamic susceptibility contrast-enhanced imaging,[4;27;58] acquisition and post-processing methods vary widely, and as yet there is no standardized protocol. Additionally, gradient-echo-planar imaging sequences suffer from susceptibility artifacts that exclude many patients from analysis.[57;61;64] Positron emission tomography with *O*-(2-[^18^F]Fluoroethyl)-l-tyrosine (FET) seems promising,[65] probably by reporting increased expression of amino acid transporters in glioma cells, but prospective evidence that treatment-related effects and recurrent glioblastoma can be distinguished is limited.[66] Furthermore, were *O*-(2-[^18^F]Fluoroethyl)-l-tyrosine positron emission tomography to be used routinely, a separate radionuclide investigation in addition to MRI would be required. The advantage of the approach described here is that it relies only on *T*_2_-weighted MR images, which are acquired routinely worldwide, are high resolution and which require no specialized pre-processing prior to image analysis (which potentially could be automated).

This study addresses several recent recommendations on imaging glioblastoma treatment response. These include that images should detect changes in tissue heterogeneity[4] and that they should cover the entire volume of pathological tissue with an acceptable slice thickness and spatial resolution[67]. Although perfusion and radionuclide imaging techniques appear promising in being able to distinguish progression from pseudoprogression, none are widely available to allow implementation in daily practice or in clinical trials. Therefore it is recommended that surrogate MRI techniques need to be developed that have the potential to be used in common clinical practice, which could make these time consuming and expensive imaging techniques obsolete.[4] It is also noteworthy that for the first time *T*_2_-weighted images have been included in the new RANO criteria, although there is as yet no detail as to how these images should be assessed.[9] If further validated prospectively in larger datasets, analysis of heterogeneity in *T*_2_-weighted images could potentially be included in future criteria for assessing treatment response.

### Strengths and limitations

A strength of this study was that the treatment given to the retrospective training cohort was uniform and reflects routine management in the UK (and many other countries). This was important for allocation of treatment response status[31] as well as survival analyses.[68] In contrast, a limitation was the small size of the prospective test cohort because of patient exclusions through insufficient imaging examinations or exposure to trial drug treatments. Small sample size of uniformly managed glioblastoma is a limitation common to much glioblastoma imaging research.[4] For example, potential external test datasets were subject to similar limitations as well as multi-centre variations in many parameters [The Cancer Genome Atlas-Glioblastoma Multiforme (TCGA-GBM) (https://wiki.cancerimaging.archive.net/display/Public/Collections); American College of Radiology (ACRIN) (http://www.acrin.org/HOME.aspx)]. Sample sizes from these alternative test datasets were similarly small because many patients did not have a complete longitudinal dataset of *T*_1_-weighted post contrast imaging, which is required for determining the treatment response based on RANO criteria, and many had been entered into drug trials or had been treated with bevacuzimab. To demonstrate that discrimination of progressors from pseudoprogressors using the SVM classifier was meaningful we performed leave-one-out cross validation on the training dataset and then tested the classifier on a separate test set, which is a robust way of preventing overfitting using machine learning methods.[69] Such an approach has been used previously to demonstrate that SVM can accurately classify clinical samples, even when used with small numbers of samples, for example *n* = 7 to 17 [70–75]. Moreover, we repeated the process with Random Forest feature selection and Lasso classification and produced the same results. There was also indirect evidence that we were not overfitting the data as the progression phenotype was concordant with the literature,[18;41;44;76–78] which was selected repeatedly by the SVM and Lasso models. Furthermore, whilst undergoing feature estimation, a difference in heterogeneity between progressors and pseudoprogressors was demonstrated unequivocally by the large divergence of progressor MFs from pseudoprogressor MFs by 7 months (Fig 2d–2f). Nonetheless, this technique might best be considered as a proof-of-concept study requiring prospective validation in a larger test dataset. Although the most significant published survival predictors[1;22–24] were incorporated as covariates during feature estimation, a larger test dataset would allow more extensive multivariate analysis.

### Conclusion

In summary, several imaging techniques appear promising in being able to distinguish progression from pseudoprogression, however, none have been validated in multicentre prospective trials or are widely available to allow implementation in daily practice or in clinical trials,[4] nor are they included in RANO recommendations.[8] We have shown here, using *T*_2_-weighted MR images alone, which are acquired routinely in the clinic, that Minkowski functionals can differentiate pseudoprogression from progression. This was achieved by capturing image information on underlying tumour heterogeneity that is only rarely visually apparent to the reporting radiologist. The key conclusion is that if this technique is further validated prospectively in larger datasets, which will probably require multi-centre studies given the relative rarity of glioblastoma, then it may be used to detect treatment response at an earlier stage, allowing an early change in treatment strategy.

## Supporting information

S1 Fig Retrospective training dataset. Modified CONSORT flow diagrams of patient inclusion and exclusion criteria for the retrospective training data set (**a**) and prospective test data set (**b**). Note that no change of therapy was apparent in all included patients. (**c**) Kaplan-Meier survival curve for patients with progression (*n* = 11) and pseudoprogression (*n* = 6). The median survival was 234 days (range 39–370) for those with progression and 585 (329–1533) for those with pseudoprogression (with one censored patient surviving).
(PDF)

S2 Fig Principal component analysis of image heterogeneity. Principal component score plot of normalized perimeter MFs from all four time points showing scans performed at 3 T (filled squares) and 1.5 T (empty squares). The modeled data were not scaled or centred. The displayed T2 Hotelling’s tolerance ellipse was set at the 0.05 significance level.
(PDF)

S1 Table Retrospective patient cohort characteristics (2005–2009). Treatment response status was significantly associated with location of the tumour and completion of adjuvant chemotherapy. As expected, the lack of completion of adjuvant chemotherapy occurred in both the progression and pseudoprogression groups predominantly because of perceived treatment failure.
(DOC)

S2 Table Linear mixed model. The results of the final linear mixed model that incorporated potential confounders. These are summarized by 2-way repeated measures ANOVA.
(DOC)

S3 Table Retrospective patient cohort characteristics (2005–2009), the training dataset. (DOC)

S4 Table Support vector machine classification. Classification of progression and pseudoprogression using a support vector machine with the training dataset.
(DOC)

S5 Table Cox proportional hazard regression analysis. Cox proportional hazard regression analysis of the three training dataset SVM models that were the most accurate.
(DOC)

S6 Table Classification applying the original SVM model to the test data set. (DOC)

S7 Table Lasso classification. Classification using a Lasso model, trained on the retrospective training dataset after Rando.
(DOC)
