# Supplementary material for: Analysis of heterogeneity in T2-weighted MR images can differentiate pseudoprogression from progression in glioblastoma
Source: PLoS One. 2017 May 17;12(5):e0176528. doi: 10.1371/journal.pone.0176528 (PMC5435159; doi:10.1371/journal.pone.0176528)
Supplement: S1 Fig — Modified CONSORT flow diagrams of patient inclusion and exclusion criteria for the retrospective training data set (a) and prospective test data set (b). Note that no change of therapy was apparent in all included patients. (c) Kaplan-Meier survival curve for patients with progression (n = 11) and pseudoprogression (n = 6). The median survival was 234 days (range 39–370) for those with progression and 585 (329–1533) for those with pseudoprogression (with one censored patient surviving). (PDF) [file pone.0176528.s001.pdf]

a

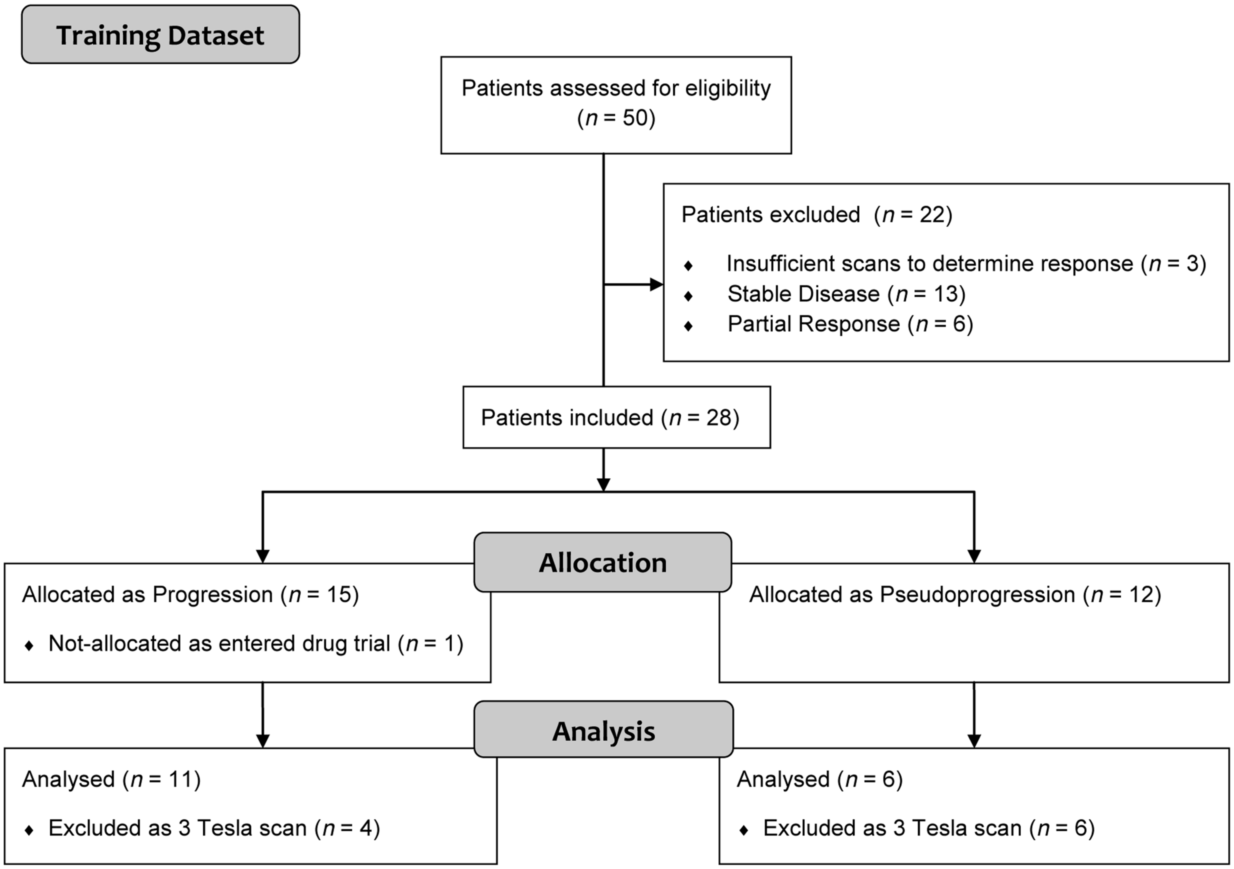
Training Dataset
Patients assessed for eligibility (n = 50)
Patients excluded (n = 22)
Insufficient scans to determine response (n = 3)
Stable Disease (n = 13)
Partial Response (n = 6)
Patients included (n = 28)
Allocation
Allocated as Progression (n = 15)
Not-allocated as entered drug trial (n = 1)
Allocated as Pseudoprogression (n = 12)
Analysis
Analysed (n = 11)
Excluded as 3 Tesla scan (n = 4)
Analysed (n = 6)
Excluded as 3 Tesla scan (n = 6)

b

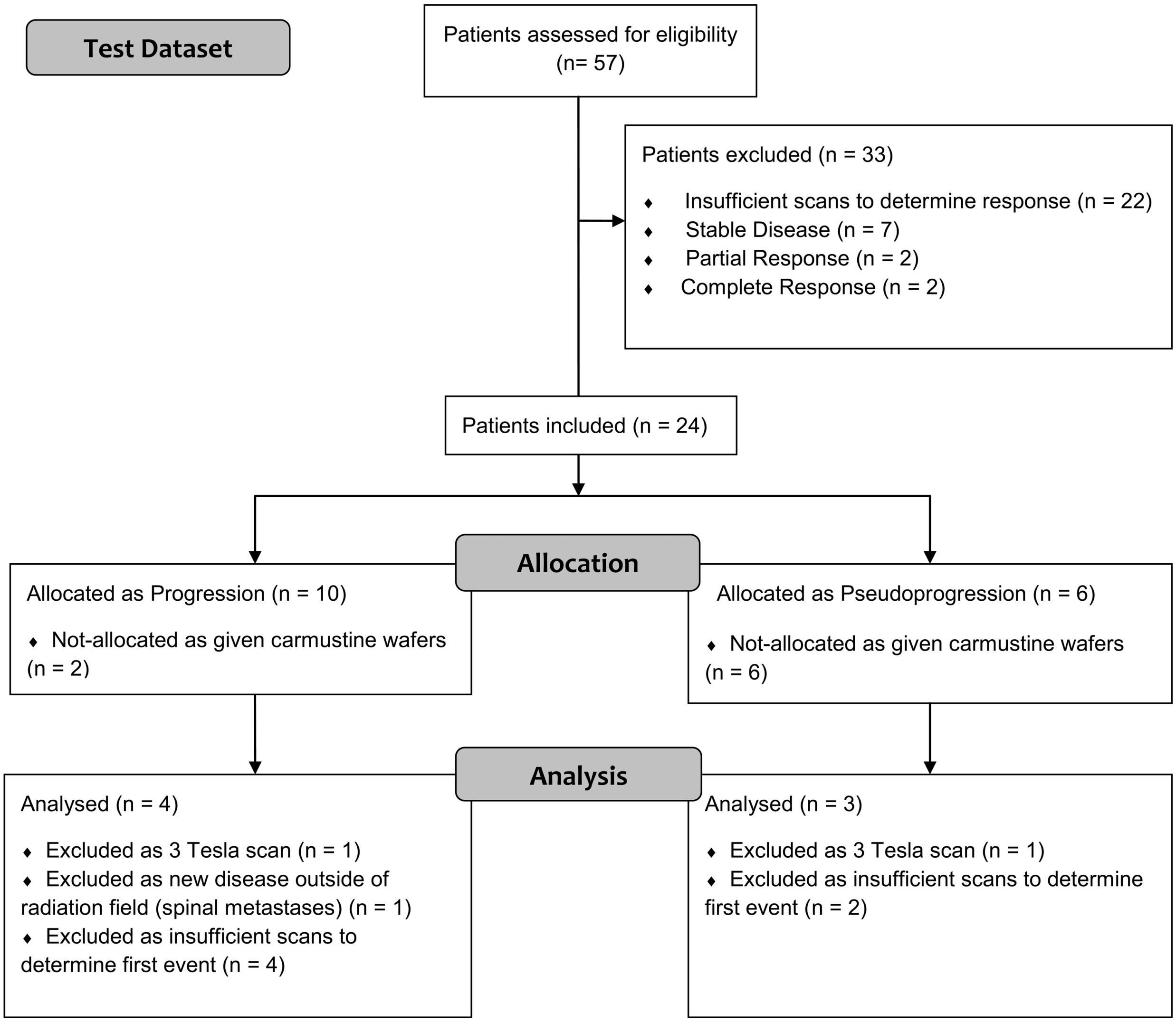
Test Dataset
Patients assessed for eligibility (n= 57)
Patients excluded (n = 33)
Insufficient scans to determine response (n = 22)
Stable Disease (n = 7)
Partial Response (n = 2)
Complete Response (n = 2)
Patients included (n = 24)
Allocation
Allocated as Progression (n = 10)
Not-allocated as given carmustine wafers (n = 2)
Allocated as Pseudoprogression (n = 6)
Not-allocated as given carmustine wafers (n = 6)
Analysis
Analysed (n = 4)
Excluded as 3 Tesla scan (n = 1)
Excluded as new disease outside of radiation field (spinal metastases) (n = 1)
Excluded as insufficient scans to determine first event (n = 4)
Analysed (n = 3)
Excluded as 3 Tesla scan (n = 1)
Excluded as insufficient scans to determine first event (n = 2)

c

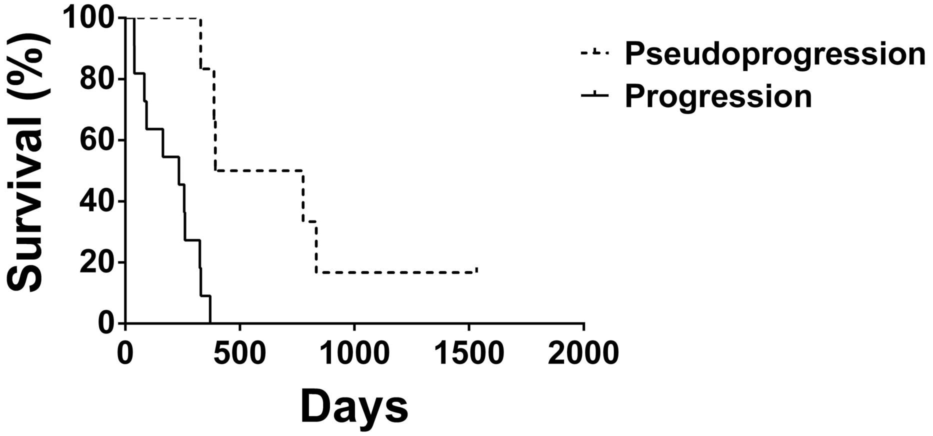

S1 Fig. Retrospective training dataset. Modified CONSORT flow diagrams of patient inclusion and exclusion criteria for the retrospective training data set (**a**) and prospective test data set (**b**). (**c**) Kaplan-Meier survival curve for patients with progression (n = 11) and pseudoprogression (n = 6). The median survival was 234 days (range 39 - 370) for those with progression and 585 (329 – 1533) for those with pseudoprogression (with one censored patient surviving).
