# Supplementary material for: Analysis of heterogeneity in T2-weighted MR images can differentiate pseudoprogression from progression in glioblastoma
Source: PLoS One. 2017 May 17;12(5):e0176528. doi: 10.1371/journal.pone.0176528 (PMC5435159; doi:10.1371/journal.pone.0176528)
Supplement: S2 Fig — Principal component score plot of normalized perimeter MFs from all four time points showing scans performed at 3 T (filled squares) and 1.5 T (empty squares). The modeled data were not scaled or centred. The displayed T2 Hotelling’s tolerance ellipse was set at the 0.05 significance level. (PDF) [file pone.0176528.s002.pdf]

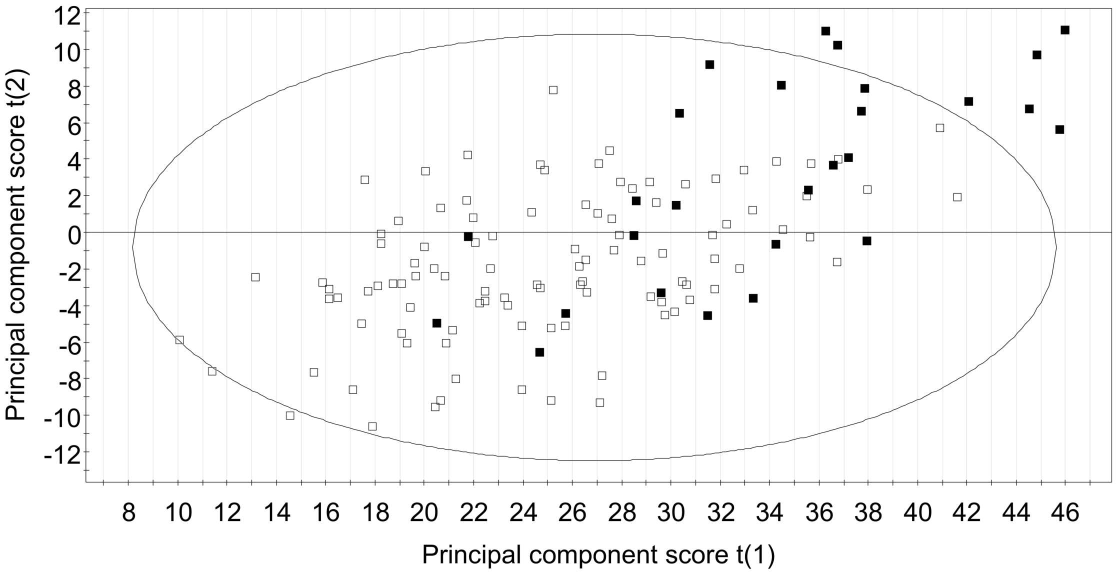

S2 Fig. Principle component analysis of image heterogeneity. Principal component score plot of normalized perimeter MFs from all four time points showing scans performed at 3 T (filled squares) and 1.5 T (empty squares). The modeled data were not scaled or centered. The displayed T2 Hotelling's tolerance ellipse was set at the 0.05 significance level.
