# Supplementary material for: Analysis of heterogeneity in T2-weighted MR images can differentiate pseudoprogression from progression in glioblastoma
Source: PLoS One. 2017 May 17;12(5):e0176528. doi: 10.1371/journal.pone.0176528 (PMC5435159; doi:10.1371/journal.pone.0176528)
Supplement: S1 Table — Treatment response status was significantly associated with location of the tumour and completion of adjuvant chemotherapy. As expected, the lack of completion of adjuvant chemotherapy occurred in both the progression and pseudoprogression groups predominantly because of perceived treatment failure. (DOC) [file pone.0176528.s003.doc]

**S1 Table. Retrospective patient cohort characteristics (2005 – 2009).** Treatment response status was significantly associated with location of the tumor and completion of adjuvant chemotherapy. As expected, the lack of completion of adjuvant chemotherapy occurred in both the progression and pseudoprogression groups predominantly because of perceived treatment failure.

| **Variable** | **Total** | **SD** | **PR** | **P** | **PsP** | **NA** | ***P*** ^a^ |
| --- | --- | --- | --- | --- | --- | --- | --- |
| **Total** | 50 | 13 | 6 | 16 | 12 | 3 |  |
| **Age, years** |  |  |  |  |  |  |  |
| **Median** | 53 | 47 | 52 | 56.5 | 53.5 | 53 | 0.09 |
| **Range** | (17 - 70) | (28 - 58) | (17 - 59) | (46 - 70) | (30 - 68) | (29 - 69) |  |
| **Karnofsy Performance Status** |  |  |  |  |  |  |  |
| **70 < 90** | 25 | 4 | 3 | 10 | 6 | 2 |  |
| **90 ≥ 100** | 25 | 9 | 3 | 6 | 6 | 1 | 0.6 |
| **Location** |  |  |  |  |  |  |  |
| **Multi-lobar** | 3 | 2 | 1 | 0 | 0 | 0 |  |
| **Single lobe** | 47 | 11 | 5 | 16 | 12 | 3 | 0.2 |
|  |  |  |  |  |  |  |  |
| **Frontal** | 11 | 3 | 3 | 3 | 2 | 0 |  |
| **Temporal** | 23 | 2 | 1 | 10 | 8 | 2 |  |
| **Parietal** | 9 | 6 | 0 | 3 | 0 | 0 |  |
| **Thalamic** | 3 | 0 | 0 | 0 | 2 | 1 |  |
| **Occipital** | 1 | 0 | 1 | 0 | 0 | 0 | 0.004 |
| **Surgery** |  |  |  |  |  |  |  |
| **Debulk** | 40 | 11 | 5 | 15 | 8 | 1 |  |
| **Biopsy** | 10 | 2 | 1 | 1 | 4 | 2 | 0.1 |
| **Temozolomide Course Completion** |  |  |  |  |  |  |  |
| **Concomitant** ^b^ | 45 | 13 | 5 | 14 | 11 | 2 | 0.3 |
| **Adjuvant** ^c^ | 27 | 12 | 5 | 3 | 5 | 2 | < 0.001 |

Abbreviations: SD, stable disease; PR, partial response; P, progression; PsP, pseudoprogression; NA, not applicable i.e. unable to assign a treatment response category; df, degrees of freedom.

^a^ Probability of obtaining a test statistic result at least as extreme as the one that was actually observed, assuming that the null hypothesis is true. Two-tailed Fisher-Freeman-Halton test used for all contingency tables. Continuous data (age) was measured with 1-way ANOVA (F = 2, df = 4); Šídák’s multiple comparison tests (all non-significant); and Brown-Forsythe test of standard deviation, which was also non-significant (*P* = 0.2, F = 2).

^b^ Incomplete due to chemotherapy complications: in PR group 1/1; P group 2/2; PsP group 1/1; NA group 1/1.

^c^ Causes for incomplete course: in SD group 1/1 due to chemotherapy complications; in PR group 1/1 due to chemotherapy complications; in P group 8/13 due to treatment failure, 2/13 due to chemotherapy complications, 3/13 due to physical deterioration; in PsP group 5/7 due to perceived treatment failure, 1/7 due to chemotherapy complications, 1/7 due to physical deterioration; in NA group 1/1 due to treatment failure.
