# Supplementary material for: Analysis of heterogeneity in T2-weighted MR images can differentiate pseudoprogression from progression in glioblastoma
Source: PLoS One. 2017 May 17;12(5):e0176528. doi: 10.1371/journal.pone.0176528 (PMC5435159; doi:10.1371/journal.pone.0176528)
Supplement: S2 Table — The results of the final linear mixed model that incorporated potential confounders. These are summarized by 2-way repeated measures ANOVA. (DOC) [file pone.0176528.s004.doc]

**S2 Table. Linear mixed model.** The results of the final linear mixed model that incorporated potential confounders. These are summarized by 2-way repeated measures ANOVA.

| **Normalized area** | **Source of variation** | | **Degrees of freedom** | | **F- Ratio** | | ***P* - Value** | |  |
| --- | --- | --- | --- | --- | --- | --- | --- | --- | --- |
|  | Intercept | | 1987 | | 3764 | | < 0.0001 | |  |
|  | Threshold | | 10490 | | 2011 | | < 0.0001 | |  |
|  | Time | | 3987 | | 8 | | < 0.0001 | |  |
|  | Tumor location | | 7 | | 3 | | 0.01 | |  |
| **Normalized Perimeter** | |  | |  | |  | |  | |
|  | | Intercept | | 1638 | | 1461 | | < 0.0001 | |
|  | | Threshold | | 10330 | | 222 | | < 0.0001 | |
|  | | Time | | 3638 | | 39 | | < 0.0001 | |
|  | | Threshold*Time | | 30638 | | 2 | | 0.01 | |
|  | | Multi-lobar | | 1 | | 8 | | 0.01 | |
|  | | Preoperative size | | 1 | | 8 | | 0.01 | |
| **Genus** |  | |  | |  | |  | |  |
|  | Intercept | | 1668 | | 392 | | < 0.0001 | |  |
|  | Threshold | | 10330 | | 35 | | < 0.0001 | |  |
|  | Time | | 3668 | | 3 | | 0.02 | |  |
|  | Preoperative size | | 1 | | 5 | | 0.04 | |  |

For normalized area the final model included fixed effect terms for threshold (*P* < 0.0001, F = 2011, 10490 df) and time (*P* < 0.0001, F = 8, 3987 df). For normalized perimeter, the final model included fixed effect terms for threshold (*P* < 0.0001, F = 222, 10330 df), time (*P* < 0.0001, F = 39, 3638 df), and the time*threshold interaction (*P* = 0.01, F = 2, 30638 df). For genus, the final model included fixed effect terms for threshold (*P* < 0.0001, F = 35, 10330 df) and time (*P* = 0.02, F = 3, 3668 df). Model checking confirmed homoscedasticity and showed that the residuals were very close to a normal distribution although there was slight kurtosis.

Other covariates contributing to the final models were less statistically significant than time and threshold. These were tumor location in the normalized area model (*P* = 0.01, F = 3, 7 df), involvement of more than one lobe (*P* = 0.01, F = 8, 1 df) in the normalized perimeter model and pre-operative size in both the normalized perimeter (*P* = 0.01, F = 8, 1 df) and genus (*P* = 0.04, F = 5, 1 df) models. The pre-operative tumor size dataset was incomplete (36/50 patients) due to the large number of patient referrals from various health systems from across the UK.
