# Supplementary material for: Analysis of heterogeneity in T2-weighted MR images can differentiate pseudoprogression from progression in glioblastoma
Source: PLoS One. 2017 May 17;12(5):e0176528. doi: 10.1371/journal.pone.0176528 (PMC5435159; doi:10.1371/journal.pone.0176528)
Supplement: S3 Table — (DOC) [file pone.0176528.s005.doc]

**S3 Table. Retrospective patient cohort characteristics (2005 – 2009), the training dataset.**

| **Variable** | **Total** | **Progression** | **Pseudoprogression** | ***P* – value** ^a^ |
| --- | --- | --- | --- | --- |
| **Number of patients** | 17 | 11 | 6 |  |
| **Age, years** |  |  |  |  |
| **Median** | 55 | 58 | 53.4 | 0.3 |
| **Range** | (30 – 68) | (46 – 62) | (30 – 68) |  |
| **Karnofsy Performance Status** |  |  |  |  |
| **70 < 90** | 10 | 6 | 4 |  |
| **90 ≥ 100** | 7 | 5 | 2 | 1 |
| **Location** |  |  |  |  |
| **Multi-lobar** | 0 | 0 | 0 |  |
| **Single lobe** | 17 | 11 | 6 | 1 |
|  |  |  |  |  |
| **Frontal** | 4 | 3 | 1 |  |
| **Temporal** | 10 | 7 | 3 |  |
| **Parietal** | 1 | 1 | 0 |  |
| **Thalamic** | 2 | 0 | 2 |  |
| **Occipital** | 0 | 0 | 0 | 0.3 |
| **Surgery** |  |  |  |  |
| **Debulk** | 13 | 10 | 3 |  |
| **Biopsy** | 4 | 1 | 3 | 0.1 |
| **Temozolomide Course Completion** |  |  |  |  |
| **Concomitant** ^b^ | 14 | 9 | 5 | 1 |
| **Adjuvant** ^c^ | 4 | 1 | 3 | 0.1 |
| **Classified on clinical grounds** | 2 | 1 | 1 | 1 |
| **First event** ^d^ |  |  |  |  |
| **4 weeks** | 13 | 8 | 5 |  |
| **4 months** | 3 | 3 | 1 | 0.5 |

^a^ Probability of obtaining a test statistic result at least as extreme as the one that was actually observed, assuming that the null hypothesis is true. 2-tailed Fisher’s exact test used for all contingency tables except for tumor location where the Fisher-Freeman-Halton test was used. Continuous data (age) was measured with the 2-tailed unpaired Student’s *t*-test (*t* = 1.1, df = 15).

^b^ Incomplete due to chemotherapy complications: in progression group 2/2; in pseudoprogression group 1/1.

^c^ Causes for incomplete course: in progression group 6/10 due to treatment failure, 2/10 due to chemotherapy complications, 2/10 due to physical deterioration; in pseudoprogression group 3/3 due to perceived treatment failure. Two progressors given salvage procarbazine, lomustine and vincristine (PCV) chemotherapy. One pseudoprogressor given salvage PCV. No patient in this dataset withdrew from the adjuvant temozolomide before the first event scan.

^d^ Time point when an enlarging, MRI-enhancing lesion occurred for the first time.
