# Supplementary material for: Analysis of heterogeneity in T2-weighted MR images can differentiate pseudoprogression from progression in glioblastoma
Source: PLoS One. 2017 May 17;12(5):e0176528. doi: 10.1371/journal.pone.0176528 (PMC5435159; doi:10.1371/journal.pone.0176528)
Supplement: S4 Table — Classification of progression and pseudoprogression using a support vector machine with the training dataset. (DOC) [file pone.0176528.s006.doc]

**S4 Table. Support vector machine classification.** Classification of progression and pseudoprogression using a support vector machine with the training dataset.

|  | **Parameters** ^a^  **___________** | | | | | | | | | | | | | | **Performance**  **____________________________________________** | | | | | | | | | | | | |
| --- | --- | --- | --- | --- | --- | --- | --- | --- | --- | --- | --- | --- | --- | --- | --- | --- | --- | --- | --- | --- | --- | --- | --- | --- | --- | --- | --- |
|  | |  | | | | | | | | |  | | | | **Trained SVM model** ^b^  **_____________________________________________** | | | | | | | | | | **LOO** ^c^  **_____** | | |
|  | | **Total C features** | | | | | | | | | **γ** | | | | **Sensitivity** | | | **Specificity** | | **PPV** | **NPV** | | **Accuracy** | | **ROC** | | |
| **Selected features** | |  | | | | | | | | |  | | | |  | | |  | |  |  | |  | | |  | |
| **Size** | |  | | | | | | | |  |  | | | |  | | |  | |  |  | |  | | |  | |
| **Features (*n*)** | | 2 | | | | | | | |  |  | | | |  | | |  | |  |  | |  | | |  | |
| **SVM parameters** | | | | | 4 | | | | | | | 2 | |  | | |  | |  | | |  | |  | | |  |
| **% (95% CI)** ^d^ | | |  | | | | |  | | | | |  | | 82 (52 - 95) | | | 83 (44 - 97) | | 90 (60 - 98) | 71 (36 - 92) | |  | |  | | |
| **% (*n*/*n*)** | | |  | | | | |  | | | | |  | | 82 (9/11) | | | 83 (5/6) | | 90 (9/10) | 71 (5/7) | | 82 (14/17) | |  | | |
| **AUC** | | |  | | | | |  | | | | |  | |  | | |  | |  |  | |  | | 0.8 | | |
| **MF** | | |  | | | | |  | | | | |  | |  | | |  | |  |  | |  | |  | | |
| **Features (*n*)** | | | 5 | | | | |  | | | | |  | |  | | |  | |  |  | |  | |  | | |
| **SVM parameters** | | | | | | 0.5 | | | | | | 1 | |  | | |  | |  | | |  | |  | | |  |
| **% (95% CI)** ^d^ | | |  | | | | |  | | | | |  | | 91 (62 - 98) | | | 83 (44 - 97) | | 91 (62 - 98) | 83 (44 - 97) | |  | |  | | |
| **% (*n*/*n*)** | | |  | | | | |  | | | | |  | | 91 (10/11) | | | 83 (5/6) | | 91 (10/11) | 83 (5/6) | | 88 (15/17) | |  | | |
| **AUC** | | |  | | | | |  | | | | |  | |  | | |  | |  |  | |  | | 0.9 | | |
| **MF & size** | | |  | | | | |  | | | | |  | |  | | |  | |  |  | |  | |  | | |
| **Features (*n*)** | | | 7 | | | | |  | | | | |  | |  | | |  | |  |  | |  | |  | | |
| **SVM parameters** | | | | | | | 1 | | | | | 1 | |  | | |  | |  | | |  | |  | | |  |
| **% (95% CI)** ^d^ | | |  | | | | |  | | | | |  | | 91 (62 - 98) | | | 83 (44 - 97) | | 91 (62 - 98) | 83 (44 - 97) | |  | |  | | |
| **% (*n*/*n*)** | | |  | | | | |  | | | | |  | | 91 (10/11) | | | 83 (5/6) | | 91 (10/11) | 83 (5/6) | | 88 (15/17) | |  | | |
| **AUC** | | |  | | | | |  | | | | |  | |  | | |  | |  |  | |  | | 0.9 | | |
| **MF & size & SI** | | | |  | | | | |  | | | |  | |  | | |  | |  |  | |  | |  | | |
| **Features (*n*)** | | | 8 | | | | |  | | | | |  | |  | | |  | |  |  | |  | |  | | |
| **SVM parameters** | | | | | | | 1 | | | | | 0.25 | | | |  |  | |  | | |  | |  | | |  |
| **% (95% CI)** ^d^ | | |  | | | | |  | | | | |  | | 91 (62 - 98) | | | 83 (44 - 97) | | 91 (62 - 98) | 83 (44 - 97) | |  | |  | | |
| **% (*n*/*n*)** | | |  | | | | |  | | | | |  | | 91 (10/11) | | | 83 (5/6) | | 91 (10/11) | 83 (5/6) | | 88 (15/17) | |  | | |
| **AUC** | | |  | | | | |  | | | | |  | |  | | |  | |  |  | |  | | 0.9 | | |

Abbreviations: CI, confidence interval (Wilson score method); LOO, leave-one-out cross-validation; PPV, positive predictive value; NPV, negative predictive value; ROC, receiver operating characteristic; AUC, area under the curve; MFs, Minkowski functionals; SI, signal intensity.

^a^ C and γ parameters were optimized through LOO grid search. The SVM was then trained using these optimized parameters.

^b^ Progression classified as progression was assigned as a true positive. Pseudoprogression classified as pseudoprogression was assigned as a true negative. Accuracy is defined as (true positive + true negative)/(true positive + false positive + true negative + false negative).

^c^ The trained model was tested using LOO to produce SVM decision values for receiver operating characteristic (ROC) analysis.

^d^ Wilson score method used to calculate confidence intervals (CI). [1]

**Reference**

1. Newcombe RG. Two-sided confidence intervals for the single proportion: comparison of seven methods. Stat Med. 1998; 17:857-872.
