# Supplementary material for: Analysis of heterogeneity in T2-weighted MR images can differentiate pseudoprogression from progression in glioblastoma
Source: PLoS One. 2017 May 17;12(5):e0176528. doi: 10.1371/journal.pone.0176528 (PMC5435159; doi:10.1371/journal.pone.0176528)
Supplement: S5 Table — Cox proportional hazard regression analysis of the three training dataset SVM models that were the most accurate. (DOC) [file pone.0176528.s007.doc]

**S5 Table. Cox proportional hazard regression analysis.** Cox proportional hazard regression analysis of the three training dataset SVM models that were the most accurate.

**Variables Regression Hazard HR Wald (χ^2^)** ^c^ ***P* > (χ^2^)** ^d^

**Selected Coefficient** ^a^ **Ratio** ^b^ **95% CI**

**SVM Model**

**Model Variables**

­­­­­­­­­­­­_________________

**MF model**

**DV MF** DV ^e^ MF 0.8 2.2 1.0 – 4.5 4.3 0.04

**Total perimeter** not selected ^f^

**Debulk status** not selected ^f^

**MF & size model**

**DV MF & size** DV ^e^ MF & size 0.7 2.0 1.1 – 3.9 4.4 0.03

**Total perimeter** not selected ^f^

**Debulk status** not selected ^f^

**MF & size & SI model**

**DV MF & size & SI** DV ^e^ MF & size & SI 0.8 1.6 1.1 – 1.8 4.4 0.04

**Total perimeter** not selected ^f^

**Debulk status** not selected ^f^

Abbreviations: DV MF, SVM decision values obtained from MFs; SI, signal intensity; CI, confidence interval; HR hazard ratio.

^a^ Coefficient examination of explanatory variable. A positive regression coefficient implies a poorer prognosis for higher values of the explanatory variable (decision values).

^b^ Hazard ratio represents the incremental increase in death per unit increase in explanatory variable (decision values).

^c^ Wald test statistic of the Cox proportional hazard model (testing that the adjusted model where covariates are fitted is significantly more powerful than the independent model where there is no impact of covariates).

^d^ Probability of obtaining a test statistic result at least as extreme as the one that was actually observed, assuming that the null hypothesis (no impact of covariates) is true.

^e^ The higher the SVM decision value, the lower the likelihood of survival

^f^ No evidence of predicting survival. The most discriminant size feature (total perimeter; Fig. 3a) and the most discriminant clinical variable (debulk status; Table S3), unlike the SVM decision values, showed no evidence of predicting survival (both variables were not selected by stepwise regression in the Cox model).
