# Supplementary material for: Analysis of heterogeneity in T2-weighted MR images can differentiate pseudoprogression from progression in glioblastoma
Source: PLoS One. 2017 May 17;12(5):e0176528. doi: 10.1371/journal.pone.0176528 (PMC5435159; doi:10.1371/journal.pone.0176528)
Supplement: S7 Table — Classification using a Lasso model, trained on the retrospective training dataset after Rando. (DOC) [file pone.0176528.s009.doc]

**S7 Table. Lasso Classification.** Classification using a Lasso model, trained on the retrospective training dataset after Random Forest selection, and then tested on the prospective test dataset.

|  | | | | **Performance** ^a^  ___________________________________________________­­­­ | | | | |  |
| --- | --- | --- | --- | --- | --- | --- | --- | --- | --- |
|  | **Total features** | | | **Sensitivity** | **Specificity** | **PPV** | **NPV** | **Accuracy** |  |
| **Selected features** |  | | |  |  |  |  |  |  |
| **MF & size** | |  |  |  |  |  |  |  |  |
| **Features (*n*)** | | 6 | |  |  |  |  |  |  |
| **% (95% CI)** ^b^ | |  |  | 100 (51 -100) | 67 (21 - 94) | 80 (38 - 96) | 100 (34 - 100) |  |  |
| **% (*n*/*n*)** | |  |  | 100 (4/4) | 67 (2/3) | 80 (4/5) | 100 (2/2) | 86 (6/7) |  |

Abbreviations: CI, confidence interval (Wilson score method); PPV, positive predictive value; NPV, negative predictive value.

^a^ Progression classified as progression was assigned as a true positive. Pseudoprogression classified as pseudoprogression was assigned as a true negative. Accuracy is defined as (true positive + true negative)/(true positive + false positive + true negative + false negative).

^b^ Wilson score method used to calculate confidence intervals (CI). [1]

**Reference**

1. Newcombe RG. Two-sided confidence intervals for the single proportion: comparison of seven methods. Stat Med. 1998; 17:857-872.
